# T Cell Responses to Nonstructural Protein 3 Distinguish Infections by Dengue and Zika Viruses

**DOI:** 10.1128/mBio.00755-18

**Published:** 2018-08-07

**Authors:** Bobby Brooke Herrera, Wen-Yang Tsai, Carlos Brites, Estela Luz, Celia Pedroso, Jan Felix Drexler, Wei-Kung Wang, Phyllis J. Kanki

**Affiliations:** a Department of Immunology and Infectious Diseases, Harvard T.H. Chan School of Public Health, Boston, Massachusetts, USA; b Department of Tropical Medicine, Medical Microbiology and Pharmacology, John A. Burns School of Medicine, University of Hawaii at Manoa, Honolulu, Hawaii, USA; c Laboratory of Infection Research, School of Medicine, Federal University of Bahia, Salvador, Brazil; d Charité Universitatsmedizin Berlin, corporate member of Freie Universität Berlin, Humboldt-Universität zu Berlin, and Berlin Institute of Health, Institute of Virology, Berlin, Germany; e German Centre for Infection Research, Germany; Tulane University Health Sciences Ctr.; Brown University

**Keywords:** Brazil, dengue virus, T cell immunity, Zika virus, nonstructural protein 3

## Abstract

The 2015–2016 Zika virus (ZIKV) epidemic in the Americas and the Caribbean demonstrated that clinical assays to detect, distinguish, and characterize immune responses to flaviviral infections are needed. ZIKV and dengue virus (DENV) are mosquito-transmitted flaviviruses sharing overlapping geographic distributions and have significant sequence similarities that can increase the potential for antibody and T cell cross-reaction. Using nonstructural protein 1-based enzyme-linked immunosorbent assays (ELISAs), we determined the serostatus of individuals living in a region of DENV and ZIKV endemicity in Brazil, identifying individuals with primary DENV (pDENV) and primary ZIKV (pZIKV), ZIKV with primary DENV (ZIKVwpDENV), and secondary DENV (sDENV) infections; the presence of pDENV and pZIKV was further confirmed by neutralization tests. Development of an enzyme-linked immunosorbent spot (ELISPOT) assay for DENV and ZIKV structural and nonstructural (NS) protein antigens enabled us to distinguish infections by these viruses based on T cell responses and to characterize those responses. We found that gamma interferon (IFN-γ) and tumor necrosis factor alpha (TNF-α) T cell responses to NS3 differentiated DENV and ZIKV infections with 94% sensitivity and 92% specificity. In general, we also showed that pDENV and sDENV cases and pZIKV and ZIKVwpDENV cases elicit similar T cell response patterns and that HIV-infected individuals show T cell responses that are lower than those shown by HIV-negative individuals. These results have important implications for DENV and ZIKV diagnostic and vaccine development and provide critical insights into the T cell response in individuals with multiple flaviviral infections.

## IMPORTANCE

The potential for antibody and T cell cross-reactions to DENV and ZIKV, flaviviruses that cocirculate and can sequentially infect individuals, has complicated diagnostic and vaccine development. Our serological data show that antibodies to nonstructural protein 1 can distinguish sequential human infections by DENV and ZIKV. The development of a simple and inexpensive assay also enables the differentiation of DENV and ZIKV infections based on characterization of T cell responses. Our T cell data reveal strong response patterns that are similar in nature to those seen with individuals with one or multiple DENV infections and with individuals with only primary ZIKV infection and ZIKV-infected individuals with previous DENV exposure. The characterization of T cell responses in a serologically validated group of individuals is of relevance to the development of vaccines and immunotherapeutics against these global threats.

## INTRODUCTION

*Aedes* mosquitoes transmit globally relevant flaviviruses, including dengue virus (DENV) and Zika virus (ZIKV). DENV exists as four antigenic serotypes, DENV1 to DENV4 (1). These viruses have a wide geographic distribution, with approximately 390 million infections annually and more than a quarter of the world’s population at risk (2). Prior to 2015, ZIKV was considered obscure and was known to circulate in Africa and Southeast Asia as two separate viral lineages, African and Asian (3). While most are asymptomatic, the clinical presentation of ZIKV infection resembles that of dengue, including fever, rash, conjunctivitis, arthralgia, and myalgia (4). In early 2015, thousands of Asian ZIKV cases appeared in northeast Brazil, with accompanying reports of severe neuropathology, including congenital microcephaly and Guillain-Barré syndrome (5, 6). In February 2016, the World Health Organization declared ZIKV a public health emergency of international concern (7). By June 2016, autochthonous transmission of ZIKV had been reported in 40 countries and in territories throughout South and Central America and the Caribbean (8).

The emergence of ZIKV in regions of DENV endemicity is of particular concern and relevant for diagnostic and vaccine development. The cocirculation of these genetically similar viruses can result in coinfection or sequential exposure, which has been shown to potentiate cross-reactive immunity at both the antibody (Ab) and T cell levels (9–12). The envelope (E) protein is the major target of the antibody response in humans during flaviviral infection (1). Antibody-based assays were found to detect extensive cross-reactivity to ZIKV E protein with other flaviviruses, requiring confirmation by plaque reduction neutralization tests (PRNTs) (11, 13–16). These tests, however, are challenged in their ability to confirm infection in individuals with multiple flaviviral infections, especially during the acute and early convalescent phases. Several studies have also shown that most DENV-immune serums or DENV E monoclonal antibodies cross-react with ZIKV but contain limited cross-neutralization activity and can instead enhance ZIKV infection, known as antibody-dependent enhancement (ADE) (17–22). In contrast, recent studies reported that antibodies to ZIKV nonstructural protein 1 (NS1) were able to discriminate infections by these viruses (23, 24). We previously showed that combinations of DENV and ZIKV NS1-based enzyme-linked immunosorbent assays (ELISAs) were capable of distinguishing confirmed cases with respect to past and present flaviviral infections, including primary DENV (pDENV) and primary ZIKV (pZIKV), ZIKV with primary DENV (ZIKVwpDENV), and secondary DENV (sDENV) infections (12). These ELISAs are applicable for routine serological tests for DENV and ZIKV and are also useful in retrospective studies to identify individuals with primary and multiple flaviviral infections.

Preexisting T cell responses to DENV have also been shown to occur with reactions to peptides encoded throughout the ZIKV proteome. DENV-naive mice challenged with ZIKV developed ZIKV-specific CD8^+^ T cells, whereas DENV-immune mice challenged with ZIKV elicited cross-reactive CD8^+^ T cells that reduced levels of infectious ZIKV (25). A study in humans infected with Asian ZIKV demonstrated that DENV serostatus influences the T cell response to ZIKV (10). DENV-immune individuals showed CD4^+^ and CD8^+^ T cell responses to ZIKV that occurred more rapidly and that were of greater magnitude than those shown by DENV-naive ZIKV-infected individuals. In addition, different patterns of immunodominant T cell responses were observed in the case of DENV and ZIKV infections. While CD8^+^ T cell responses against DENV target nonstructural (NS) proteins such as NS3, NS4B, and NS5, ZIKV-specific CD8^+^ T cell responses target the capsid (C), premembrane (prM), and E structural proteins (10, 26). We previously developed a modified anthrax toxin (N-terminal domain of lethal factor [LFn])-based enzyme-linked immunosorbent spot (ELISPOT) assay, which revealed long-term T cell responses that were ZIKV and DENV specific with respect to NS3 protease but cross-reactive with respect to NS3 helicase in individuals infected with DENV and African ZIKV (27). The impact of cross-reactive immune responses in protection or development of ZIKV-mediated neuropathology remains unclear.

In this study, we utilized our NS1-based ELISAs to determine the DENV and ZIKV serostatus of individuals from Salvador, Brazil, a region of DENV hyperendemicity with one of the highest incidence rates of ZIKV during the 2015–2016 epidemic (28). We then tested the ability of our LFn ELISPOT test to distinguish infections by DENV and Asian ZIKV based on T cells and to characterize those responses.

## RESULTS

### NS1-based ELISAs and neutralization tests determine DENV and ZIKV serostatus.

During the ZIKV outbreak in Salvador, Brazil, acute-phase blood samples were collected from hundreds of suspected ZIKV-infected patients attending HIV outpatient clinics between November 2015 and May 2016. Serological testing for ZIKV-NS1 IgG and DENV-E IgG was performed, revealing a high incidence of ZIKV infection in presumed DENV-immune and -naïve individuals (28). Fifty of these patients were included in the present study. Their median age was 43 (range, 23 to 72), 49% were female, and 76% were infected with human immunodeficiency virus (HIV). All HIV-infected individuals were on antiretroviral therapy; more than 92% had undetectable viral loads and normal CD4 counts. Data pertaining to acute serology and patient characteristics are summarized in Table 1 and Table S1, respectively.

10.1128/mBio.00755-18.1 TABLE S1  Cohort characteristics. Download TABLE S1, DOCX file, 0.03 MB. Copyright © 2018 Herrera et al. 2018 Herrera et al. This content is distributed under the terms of the Creative Commons Attribution 4.0 International license.

**TABLE 1:** Results of serological tests^g^

ID	Acute-phase sera			Late-convalescent-phase serum						Interpretation
	ZIKV-NS1 IgG^a^	DENV-E IgG^a^	PRNT test^b^	ZIKV-NS1 IgG^c^	DENV-NS1 IgG^c^	rOD ratio^c^	ZIKV-E IgG^d^	DENV-E IgG^d^	NT test results for D1/D2/D3/D4/ZIKV^f^	
ZK0978	−	−	−	−	−	NA	−	−	<10/<10/<10/<10/<10	Negative
ZK0982	−	−	−	−	−	NA	−	−	<10/<10/<10/<10/<10	Negative
ZK0987	−	−	−	−	−	NA	−	−	<10/<10/<10/<10/<10	Negative
ZK0999	−	−	−	−	−	NA	−	−	<10/<10/<10/<10/<10	Negative
ZK0979	+	+		+	−	NA	+^e^	+	<10/<10/<10/<10/>160	pZIKV
ZK0993	+	+		+	−	NA	+^e^	+	<40/<40/<10/<10/>160	pZIKV
ZK0998	+	+		+	−	NA	+^e^	+	<10/<10/<40/<10/>160	pZIKV
ZK1006	+	+		+	−	NA	+^e^	+	<80/<40<10/<40/640	pZIKV
ZK0996	+	−	+	+	−	NA	+^e^	+	<10/<10/<10/<10/>160	pZIKV
ZK0966	−	+	−	−	−	NA	+	+^e^	<40/160/<10/<40/<10	pDENV
ZK0980	−	+		−	−	NA	+	+^e^	<40/<40/>160/<40/<10	pDENV
ZK0995	−	+		−	−	NA	+	+^e^	>640/160/<10/<10/<10	pDENV
ZK0997	ND	ND		−	−	NA	+	+^e^	<40/>160/<40/<40/<10	pDENV
ZK0972	+	+		+	+	≥0.24	+	+	>40/ND/ND/ND/>80	ZIKVwpDENV
ZK0975	+	+		+	+	≥0.24	+	+	>40/ND/ND/ND/>80	ZIKVwpDENV
ZK0989	+	+	+	+	+	≥0.24	+	+	>40/ND/ND/ND/>80	ZIKVwpDENV
ZK0991	+	+	+	+	+	≥0.24	+	+	>40/ND/ND/ND/>80	ZIKVwpDENV
ZK1000	+	+		+	+	≥0.24	+	+	>40/ND/ND/ND/>40	ZIKVwpDENV
ZK1009	+	+	+	+	+	≥0.24	+	+	>10/ND/ND/ND/>80	ZIKVwpDENV
ZK1011	+	+		+	+	≥0.24	+	+	>40/ND/ND/ND/>80	ZIKVwpDENV
ZK1012	+	+	+	+	+	≥0.24	+	+	>40/ND/ND/ND/>80	ZIKVwpDENV
ZK1014	+	+	+	+	+	≥0.24	+	+	>40/ND/ND/ND/>80	ZIKVwpDENV
ZK1015	+	+	+	+	+	≥0.24	+	+	>40/ND/ND/ND/>80	ZIKVwpDENV
ZK0968	+	ND		+	+	≥0.24	+	+	>40/ND/ND/ND/>80	ZIKVwpDENV
ZK0984	+	+		+	+	≥0.24	+	+	>320/>1,280/<80/>1,280/>320	ZIKVwpDENV
ZK0976	−	+		+	+	<0.24	+	+	>40/ND/ND/ND/<10	sDENV
ZK0986	−	+		+	+	<0.24	+	+	>40/ND/ND/ND/>80	sDENV
ZK0967	+	+		+	+	<0.24	+	+	>40/ND/ND/ND/>80	sDENV
ZK0969	+	+		+	+	<0.24	+	+	>40/ND/ND/ND/>40	sDENV
ZK0971	+	+		+	+	<0.24	+	+	>40/>40/ND/ND/<10	sDENV
ZK0977	+	+		+	+	<0.24	+	+	>40/ND/ND/ND/>80	sDENV
ZK0983	+	+	+	+	+	<0.24	+	+	>40/ND/ND/ND/>10	sDENV
ZL0985	+	+	+	+	+	<0.24	+	+	>40/ND/ND/ND/>40	sDENV
ZK0988	+	+		+	+	<0.24	+	+	>40/ND/ND/ND/>40	sDENV
ZK0992	+	+	+	+	+	<0.24	+	+	>40/ND/ND/ND/>80	sDENV
ZK0994	+	+	−	+	+	<0.24	+	+	>40/ND/ND/ND/>40	sDENV
ZK1001	+	+		+	+	<0.24	+	+	>40/>40/ND/ND/<10	sDENV
ZK1010	+	+		+	+	<0.24	+	+	>40/ND/ND/ND/>80	sDENV
ZK1013	+	ND		+	+	<0.24	+	+	>40/ND/ND/ND/>10	sDENV
ZK0973	−	ND		−	+	<0.24	+	+	>40/<80/>80/ND/>320	sDENV
ZK0974	−	+		−	+	<0.24	+	+	>40/>320/>80/ND/>10	sDENV
ZK1002	−	+	−	−	+	<0.24	+	+	>40/>80/<80/ND/<10	sDENV
ZK1003	−	+	−	−	+	<0.24	+	+	>40/>80/<80/ND/<10	sDENV
ZK1007	−	+		−	+	<0.24	+	+	>40/>80/>80/ND/<10	sDENV
ZK1016	−	+		−	+	<0.24	+	+	>40/>1,280/>1,280/ND/>10	sDENV
ZK0990	−	+	−	−	+	<0.24	+	+	>40/>1,280/>80/ND/<10	sDENV
ZK1004	+	+		−	+	NA	+	+	ND/ND/ND/ND/ND	Unknown
ZK1005	+	ND		−	+	NA	+	+	ND/ND/ND/ND/ND	Unknown
ZK1008	+	+		−	−	NA	+	+	ND/ND/ND/ND/ND	Unknown
ZK0981	−	ND		−	−	NA	−	−	ND/ND/ND/ND/ND	Unknown

a Euroimmun ZIKV-NS1 and DENV-E IgG ELISAs were performed on acute-phase sera (28).

b PRNT was performed on acute-phase sera to detect neutralization antibody to ZIKV (47).

c ZIKV-NS1 and DENV-NS IgG ELISAs were described previously (12). rOD ratio (ZIKV-NS1/DENV-NS1) values of <0.24 or ≥0.24 were classified as representative of sDENV or ZIKVwpDENV infection, respectively (12).

d ZIKV-E and DENV-E IgG ELISAs utilized ZIKV VLP and DENV virions, respectively (46). ΔrOD (rOD of ZIKV − rOD of DENV) values of greater than or equal to 0.17 or less than −0.17 were classified as representative of pZIKV or pDENV infection, respectively.

e ZIKV-E and DENV-E IgG ELISAs utilized ZIKV VLP and DENV virions, respectively (46). ΔrOD (rOD of ZIKV − rOD of DENV) values of greater than or equal to 0.17 or less than −0.17 were classified as representative of pZIKV or pDENV infection, respectively.

f Microneutralization tests (NT) were performed (NT_90_ titers are shown) to confirm no infection or pZIKV or pDENV infection (46, 48).

g pDENV, primary DENV infection; pZIKV, primary ZIKV infection; sDENV, secondary DENV infection; ZIKVwpDENV, ZIKV infection with previous DENV infection. D1, DENV serotype 1; D2, DENV serotype 2; D3, DENV serotype 3; D4, DENV serotype 4; NA, not applicable; ND, not determined.

**FIG 1:**
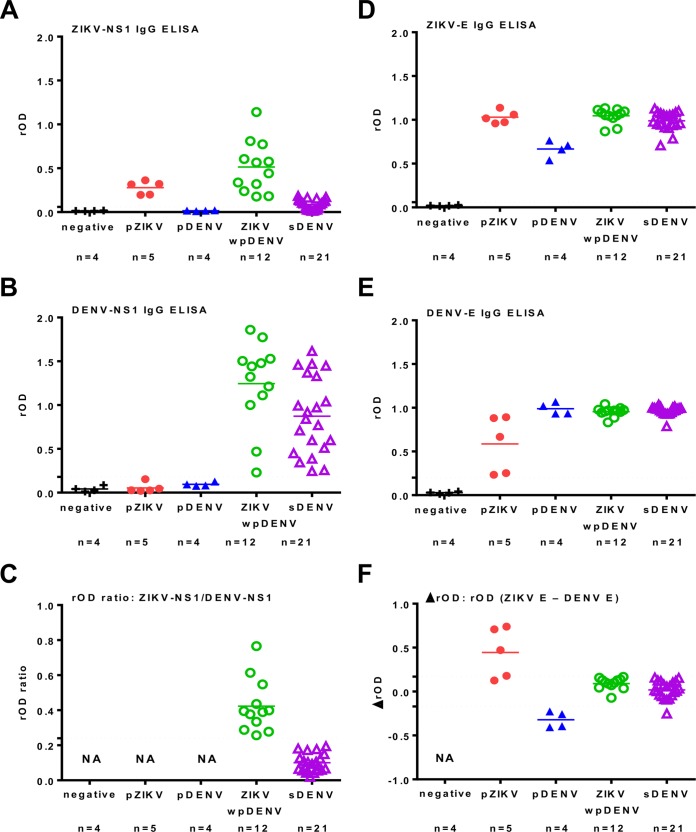
ZIKV and DENV NS1-based and E-based IgG ELISAs. (A) ZIKV-NS1 IgG ELISA. (B) DENV-NS1 IgG ELISA. (C) ZIKV-NS1/DENV-NS1 rOD ratio. (D) ZIKV-E IgG ELISA. (E) DENV-E IgG ELISA. (F) ΔrOD = rOD of ZIKV − rOD of DENV. Horizontal lines indicate cutoff values (0.24 for rOD ratio and 0.17 for ΔrOD). pZIKV, primary ZIKV infection; pDENV, primary DENV infection; sDENV, secondary DENV infection; ZIKVwpDENV, ZIKV infection with previous DENV infection. NA, not applicable.

In order to determine the DENV and ZIKV serostatus among the study participants who had potentially been dually exposed, we collected late-convalescent-phase blood samples and employed our previously developed ZIKV-NS1 and DENV-NS1 IgG ELISAs (22). For samples positive for DENV-NS1, we calculated the ratio of relative optical density (rOD) of ZIKV-NS1 to that of DENV-NS1 and used a rOD ratio of <0.24 or ≥0.24 to determine sDENV or ZIKVwpDENV infection, respectively (22). Twelve ZIKVwpDENV infections and 21 sDENV infections were identified (Table 1) (Fig. 1A to C). Five samples with ZIKV-NS1-positive and DENV-NS1-negative results represented pZIKV. Since these samples were collected more than 1 year postinfection, some anti-NS1 antibodies may have declined to levels below detection, so we further tested with ZIKV and DENV E protein-based ELISAs and identified four samples negative for both ZIKV and DENV in all four ELISAs tested (Table 1). Based on the difference in rOD of ZIKV and DENV E proteins (ΔrOD = rOD of ZIKV − rOD of DENV), we identified four pZIKV infections (ΔrOD greater than or equal to 0.17) and four pDENV infections (ΔrOD less than −0.17) (Table 1) (Fig. 1D and E). The negative pZIKV and pDENV samples were further confirmed by microneutralization tests of DENV1 to DENV4 and ZIKV; all four negative samples had 90% neutralization (NT_90_) titers of <10 for DENV1 to DENV4 and ZIKV, and the five pZIKV and four pDENV samples showed monotypic neutralization patterns for ZIKV and for one of the four DENV serotypes, respectively (Table 1). For the remaining 33 samples, microneutralization tests were performed with ZIKV, DENV1, and/or DENV2 or DENV3 to show that all 12 ZIKVwpDENV samples neutralized (NT_90_ titers of ≥10) ZIKV plus at least one DENV serotype, whereas all 21 sDENV samples neutralized at least two DENV serotypes or DENV plus ZIKV; these patterns were compatible with unspecified flavivirus infection according to CDC guidelines (16). An additional three samples (ZK1004, ZK1005, and ZK1008), which showed ΔrOD values between −0.17 and 0.17 and positive ZIKV-NS1 IgG results for acute-phase sera but negative results for late-convalescent-phase sera, were classified as undetermined (Table 1). Another sample (ZK0981), for which DENV acute-phase serology was not performed, was negative for ELISAs using late-convalescent-phase serum and was also classified as undetermined.

### T cell responses to NS3 distinguish DENV and ZIKV infections.

**FIG 2:**
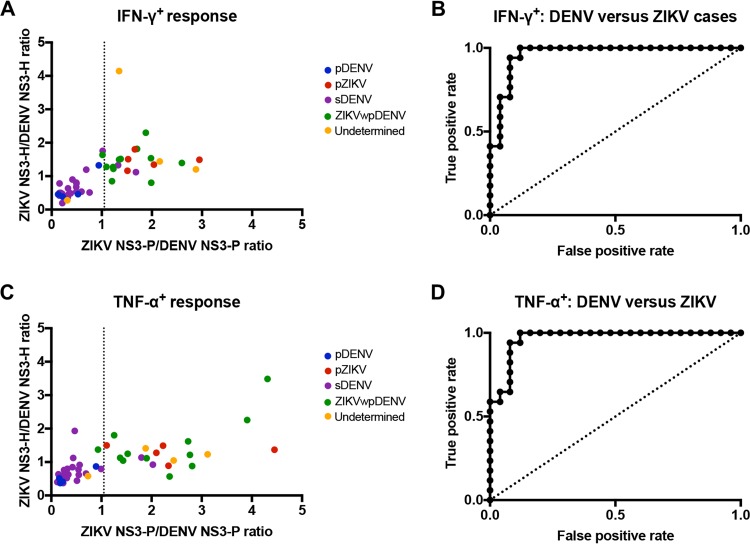
T cell responses to NS3 protease and helicase and ROC analysis of the ELISPOT test. Late-convalescent-phase PBMCs from DENV-infected and/or ZIKV-infected individuals were treated with homologous and heterologous LFn-DENV and LFn-ZIKV NS3 protease, and the specific IFN-γ and TNF-α T cell responses were detected by *ex vivo* ELISPOT tests. (A) Scatter plot of the ratios of ZIKV NS3 protease to DENV NS3 protease IFN-γ responses versus ratios of helicase. (B) ROC analysis of the IFN-γ ELISPOT test. (C) Scatter plot of the ratios of ZIKV NS3 protease to DENV NS3 protease TNF-α T cell responses versus ratios of helicase responses. (D) ROC analysis of the TNF-α ELISPOT test. The dashed line in panel A represents the optimal cutoff value of 1.05, and the dashed line in panel C represents the optimal cutoff value of 1.048. Individual colored dots represent serologically validated DENV-infected and/or ZIKV-infected individuals and the undetermined cases.

We recently reported the development of an LFn ELISPOT test based on NS3 protease and helicase to distinguish DENV and African ZIKV human infections (27). To assess the ability of the assay to distinguish infections by DENV and Asian ZIKV, we performed DENV and ZIKV homologous and heterologous LFn-NS3 protease and helicase stimulation of date-matched late-convalescent-phase peripheral blood mononuclear cells (PBMCs) in separate gamma interferon (IFN-γ) and tumor necrosis alpha (TNF-α) ELISPOT tests among the serological-validated pDENV, pZIKV, sDENV, ZIKVwpDENV, and undetermined cases. Using an NS3 protease-to-helicase-ratio cutoff value of 1.05 for the IFN-γ ELISPOT test, pDENV and sDENV cases and pZIKV and ZIKVwpDENV and 3 of the 4 serologically undetermined cases appeared to group together (Fig. 2A). Among the undetermined cases, 3 (ZK1004, 1005, and 1008) of 4 grouped with the ZIKV-exposed individuals, while ZK0981 grouped with the DENV-infected individuals. Using a ratio cutoff value of 1.048 for the TNF-α ELISPOT test, similar groupings were observed (Fig. 2B). We were unable to distinguish sequential infections based on T cell responses to NS3 protease and helicase.

Test data were further analyzed to define sensitivity (identifying true positives, i.e., individuals who had been infected by DENV versus ZIKV) and specificity (identifying true negatives, i.e., DENV- or ZIKV-uninfected individuals). We evaluated sensitivity and specificity as functions of the IFN-γ and TNF-α cutoff values, above which a sample was considered positive and below which a sample was considered negative. We grouped pDENV and sDENV cases and pZIKV and ZIKVwpDENV cases based on the clustering observed and excluded the 4 serologically undetermined cases from the analysis. Receiver operating characteristic (ROC) curves and corresponding numerical values illustrate the performance of the ELISPOT tests as a function of the discrimination threshold, plotted as sensitivity versus 1 − specificity. The areas of the ROC curves represent test performance, where 1 represents a perfect test and 0.5 a random predictor. We measured areas of 0.96 and 0.97 for the IFN-γ and TNF-α ELISPOT tests, respectively (Table 2) (Fig. 2C and D). Using the cutoff values, the test sensitivity and specificity for both the IFN-γ and TNF-α ELISPOT tests were 94% and 92%, respectively.

**TABLE 2:** Numerical values of ROC and sensitivity and specificity analysis results

Parameter	Value(s) for DENV versus ZIKV	
	IFN-γ^+^	TNF-α^+^
AUC^a^	0.96	0.97
95% CI^b^	0.91–1.02	0.92–1.01
Cutoff	1.055	1.048
% sensitivity	94	94
% specificity	92	92

a AUC, area under the curve.

b CI, confidence interval.

### LFn-DENV and LFn-ZIKV structural and nonstructural proteins elicit robust T cell responses, and prior DENV exposure does not affect the response.

**FIG 3:**
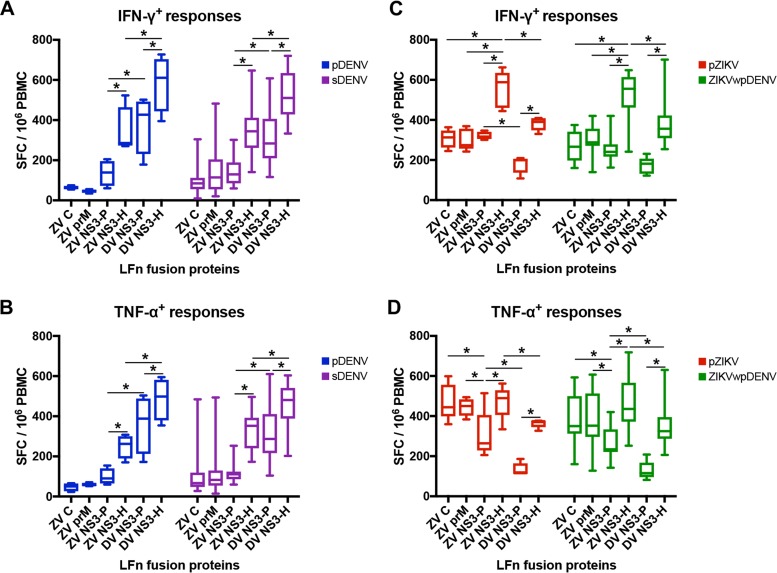
T cell responses to ZIKV and/or DENV structural or nonstructural proteins among subgroups with different DENV and ZIKV serostatus. Late-convalescent-phase PBMCs from DENV-infected and/or ZIKV-infected individuals were treated with homologous and/or heterologous LFn-DENV and LFn-ZIKV capsid (ZV C), premembrane (ZV prM), NS3 protease (DV or ZV NS3-P), and NS3 helicase (DV or ZV NS3-H), and the specific IFN-γ and TNF-α T cell responses were detected by *ex vivo* ELISPOT tests. IFN-γ and TNF-α spot-forming cells (SFC) were detected and counted, and the results were expressed as box plots with means and standard deviations. (A and B) Comparisons of late-convalescent-phase IFN-γ (A) and TNF-α (B) T cell responses of individuals with pDENV and sDENV infections. (C and D) Comparisons of late-convalescent-phase IFN-γ (C) and TNF-α (D) T cell responses of individuals with pZIKV and ZIKVwpDENV infections. Individual colored plots represent serologically validated DENV-infected and/or ZIKV-infected individual. *, *P* < 0.05.

To assess the magnitude of T cell responses among the study participants, we stimulated late-convalescent-phase PBMCs in separate IFN-γ and TNF-α ELISPOT tests using the following six LFn fusion proteins: LFn-DENV-NS3-protease (LFn-DV NS3-P), LFn-DENV-NS3-helicase (LFn-DV NS3-H), LFn-ZIKV-capsid (LFn-ZV C), LFn-ZIKV-premembrance (LFn-ZV prM), LFn-ZIKV-NS3-protease (LFn-ZV NS3-P), and LFn-ZIKV-NS3-helicase (LFn-ZV NS3-H). Individuals with pDENV and sDENV infections showed similar IFN-γ and TNF-α T cell response patterns (Fig. 3A and B). These individuals had T cell responses to LFn-DV NS3-H and LFn-ZV NS3-H that were greater in magnitude than the responses to LFn-DV NS3-P and LFn-ZV NS3-P, respectively. Additionally, T cell responses to LFn-DV NS3-P and NS3-H were stronger than those seen with LFn-ZV NS3-P and NS3-H. The amount of T cell cross-reactivity to the ZIKV structural proteins (LFn-ZV C and LFn-ZV prM) was limited compared to the high cross-reactivity to LFn-ZV NS3-P and NS3-H. Furthermore, individuals with pZIKV and ZIKVwpDENV infections showed T cell responses to LFn-ZV NS3-H and LFn-DV NS3-H that were greater in magnitude than those seen with LFn-ZV NS3-P and LFn-DV NS3-P, respectively (Fig. 3C and D). While individuals with pZIKV and ZIKVwpDENV infections had stronger IFN-γ T cell responses to LFn-ZV NS3-H than to the ZIKV structural proteins, TNF-α responses to the ZIKV structural proteins were stronger than to LFn-ZV NS3-P.

We further evaluated the impact of DENV immunity on the magnitude of T cell responses. We compared the magnitudes of the IFN-γ and TNF-α T cell responses of individuals with pDENV and sDENV infections and of individuals with pZIKV and ZIKVwpDENV infections. In all cases, the T cell responses in individuals with prior DENV exposure were not significantly higher than those seen in individuals with a primary DENV or ZIKV infection (Fig. 3). While IFN-γ and TNF-α responses to the ZIKV structural proteins in individuals with sDENV infections appeared stronger than the responses to those in individuals with pDENV infections, these differences were not statistically significant. Similarly, individuals with ZIKVwpDENV infection had IFN-γ and TNF-α T responses comparable to those of individuals with pZIKV infections (Fig. 3C and D).

### HIV influences the T cell response in DENV-exposed individuals.

**FIG 4:**
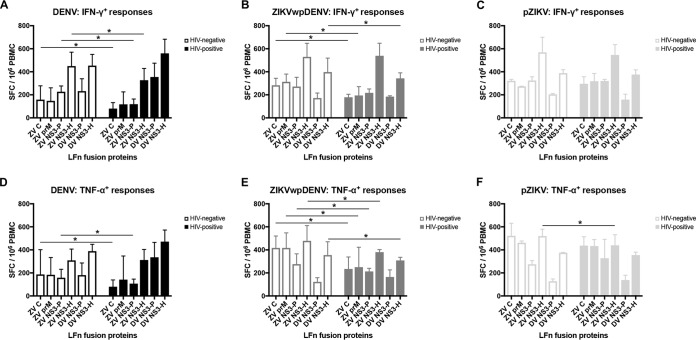
Impact of HIV status on the T cell response. (A to C) Comparisons of mean convalescent-phase IFN-γ T cell responses expressed as bars and standard deviations between HIV-negative (open black bars) and HIV-infected (shaded black bars) individuals with pDENV and sDENV infections grouped together (A), HIV-negative (open dark gray bars) and HIV-infected (shaded dark gray bars) individuals with ZIKVwpDENV infections (B), and HIV-negative (open light-gray bars) and HIV-infected (shaded light-gray bars) individuals with pZIKV infections (C). (D to F) Comparisons of mean convalescent-phase TNF-α T cell responses indicated as bars and standard deviations between (D) HIV-negative (open black bars) and HIV-infected (shaded black bars) individuals with pDENV and sDENV infections grouped together, (E) HIV-negative (open dark gray bars) and HIV-infected (shaded dark gray bars) individuals with ZIKVwpDENV infections, and (F) HIV-negative (open light-gray bars) and HIV-infected (shaded light-gray bars) individuals with pZIKV infections. *, *P* < 0.05.

We also compared the magnitudes of the IFN-γ and TNF-α T cell responses in DENV-exposed (grouping individuals with pDENV and sDENV infections together), pZIKV-infected, and ZIKVwpDENV-infected individuals who were HIV negative or HIV infected. DENV-exposed HIV-negative individuals had stronger IFN-γ responses to LFn-ZV C, LFn-ZV NS3-P, and LFn-ZV NS3-H than HIV-infected individuals. IFN-γ responses to LFn-DV NS3-P and NS3-H appeared to be stronger in the HIV-infected individuals, although these differences were not statistically significant (*P* = 0.61 and *P* = 0.13, respectively) (Fig. 4A). A similar pattern of responses was observed for TNF-α (Fig. 4D). In general, ZIKVwpDENV HIV-negative individuals had stronger IFN-γ and TNF-α responses than individuals that were HIV infected (Fig. 4B and E). In contrast, there was largely no difference in the IFN-γ and TNF-α responses in pZIKV HIV-negative and HIV-infected individuals (Fig. 4C and F). There was an exception where the TNF-α response to LFn-ZV NS3-H was stronger in individuals that were HIV negative.

## DISCUSSION

We report on the characterization of late-convalescent-phase antibody and T cell responses in individuals from Salvador, Brazil, a region of DENV hyperendemicity that was burdened by the 2015–2016 ZIKV outbreak. Our report presents three major findings in a serologically validated group of DENV-infected and/or ZIKV-infected individuals. First, IFN-γ and TNF-α T cell response ratios of ZIKV NS3 protease to DENV NS3 protease can discriminate infections in individuals exposed to these viruses. Second, individuals with pDENV and sDENV infections have similar T cell response patterns, with extensive cross-reactivity to ZIKV NS3 helicase, whereas individuals with pZIKV and ZIKVwpDENV infections have strong responses to both ZIKV structural and nonstructural proteins, with high cross-reaction to DENV NS3 helicase. Third, HIV infection is associated with responses that are lower in DENV-exposed individuals.

Our previous study of NS1-based ELISAs of convalescent-phase sera from reverse transcription-PCR (RT-PCR)-confirmed cases with pZIKV, pDENV, sDENV, and ZIKVwpDENV infections showed that sDENV infection panels cross-react to ZIKV-NS1 and that the rOD ratio of ZIKV-NS1 to DENV-NS1 in IgG ELISA can distinguish sDENV infections from ZIKVwpDENV infections (22). Since levels of anti-NS1 antibodies may decline over time and become undetectable, especially for those with primary infection, we further tested these samples with E protein-based IgG ELISAs and identified four negative samples and five pZIKV and four pDENV infections. All 13 of those samples have been verified by neutralization tests using an NT_90_ value of ≥10 as the cutoff based on the CDC guidelines (16), suggesting that ΔrOD values based on ZIKV-E and DENV-E IgG ELISAs can distinguish pZIKV and pDENV infections; this could potentially be a useful tool for epidemiology and pathogenesis study in regions of endemicity. However, the sample size was small and the ΔrOD of 0.17 was based on a single serum dilution of 1:800; future studies involving larger sample size and different dilutions or end point titers are needed to further validate these observations.

**FIG 5:**
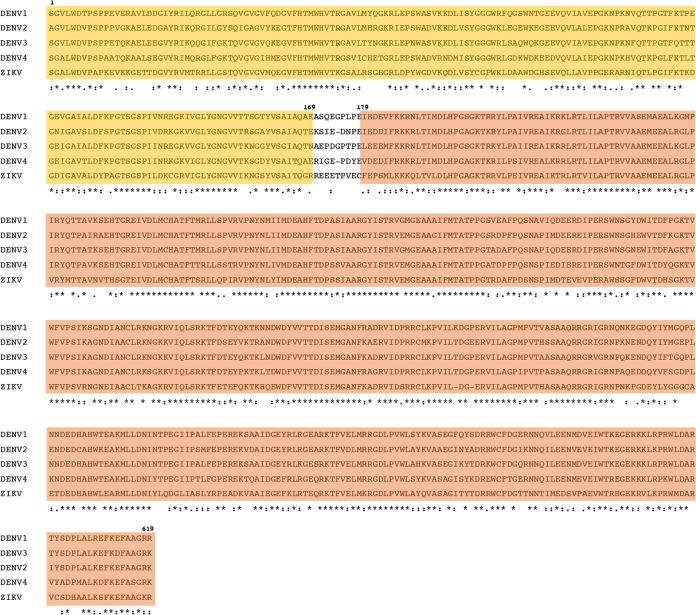
Clustal Omega-generated amino acid sequence alignment of DENV serotypes 1 to 4 and Asian ZIKV. The residues in yellow represent the NS3 protease domain (amino acids 1 to 169), and the residues in orange represent the helicase domain (amino acids 179 to 619). *, single, fully conserved residue; :, conservation between groups of strongly similar properties (i.e., those scoring >0.5 in the Gonnet PAM 250 matrix); •, conservation between groups of weakly similar properties (i.e., those scoring ≤0.5 in the Gonnet PAM 250 matrix).

The proportions of amino acid sequence identity between DENV and ZIKV structural and nonstructural proteins are 49% and 51%, respectively (10). Multiple-sequence alignment and DENV and ZIKV NS3 homology determination results demonstrate a high amino acid sequence identity of 67%, with protease and helicase homologies of 58% and 72%, respectively, consistent with the higher degree of DENV/ZIKV cross-reaction in NS3 helicase (Table 3) (Fig. 5). Our recent characterization of acute- and convalescent-phase T cells collected from individuals infected with DENV and African ZIKV in Senegal, West Africa, revealed sustained DENV- and ZIKV-specific responses to NS3 protease and cross-reactive responses to NS3 helicase (27). Our findings in individuals infected with DENV and Asian ZIKV are in agreement with our previous observations. Although we were unable to distinguish sequential exposures, the LFn NS3 protease ELISPOT test differentiates between infections in DENV- and ZIKV-infected individuals with high sensitivity and specificity of 94% and 92%, respectively.

**TABLE 3:** Sequence homology of DENV and Asian ZIKV NS3^a^

Serotype	% homology to ZIKV		
	NS3 protease	NS3 helicase	Full-length
DENV1	55	71	66
DENV2	58	72	67
DENV3	58	72	67
DENV4	59	71	67

a Data represent results of homology analyses of comparisons of Asian ZIKV (GenBank accession number NC_035889.1) to DENV1 (ACO06157.1), DENV2 (JN819419.1), DENV3 (ACY70771.1), and DENV4 (AEW50183.1).

A relatively large body of epidemiological and laboratory-based evidence has suggested that severe and often fatal forms of dengue disease occur most commonly during a secondary infection by a heterotypic DENV serotype (29, 30). Another phenomenon, known as original antigenic sin (OAS), has been observed in antibody as well as T cell responses, in which less-effective T cells generated in response to a primary DENV infection predominate during a subsequent infection with a different DENV serotype, resulting in an inappropriate response and predisposing individuals to severe disease (31, 32). The OAS hypothesis was challenged by a study in Sri Lankan individuals infected with DENV, which showed that the phenomenon does not generate less-functional responses but instead correlates with protective responses to conserved viral sequences (26). Unexpectedly, we did not observe T cell responses that were significantly higher in individuals with prior DENV exposure. These results are in contrast to our data on African ZIKV infections, which showed that previous flavivirus exposure was associated with enhanced T cell responses (27). One possibility is that the proportion of HIV infection among those with prior DENV exposure was higher than that seen with DENV-naive individuals in this study (90.5% versus 50% comparing sDENV and pDENV cases; 83.3% versus 60% comparing ZIKVwpDENV and pZIKV cases). Nevertheless, as cocirculation of DENV, ZIKV, and other flaviviruses occurs throughout many parts of the world, it is critical to continue to develop tools to better understand T cell immunity in individuals exposed to multiple flaviviruses.

A recent study using human leucocyte antigen (HLA) transgenic mice infected with DENV2 and Asian and African ZIKV strains revealed cross-reactive T cell responses to HLA-restricted epitopes (25). Of 8 ZIKV NS3 epitopes computationally predicted to bind HLA class I molecules, only 3 epitopes elicited DENV2/Asian ZIKV cross-reactive T cell responses. Of note, the cross-reactive epitopes were all positioned within the helicase domain of NS3, further supporting our observations of high DENV/ZIKV NS3 helicase T cell cross-reaction. Another study demonstrated ZIKV-specific and ZIKV/DENV cross-reactive T cell responses in humans (10). T cell responses generated in response to prior DENV exposure recognized peptides sequences located throughout the ZIKV proteome. DENV serostatus also influenced T cell immunity to ZIKV. DENV-naive ZIKV-positive individuals had predominant CD8 T cell responses directed against structural proteins. In contrast, a majority of CD8 T cells responses were directed against nonstructural proteins in DENV-immune ZIKV-positive individuals, suggesting that previous DENV exposure can alter the T cell response.

While the studies cited above used peptide stimulation to characterize the T cell response, there are concerns regarding this approach (33). Some of the HLA-predicted peptides may fail to stimulate the expected strong T cell responses. Longer and shorter peptides have also been shown to elicit different types of responses (34–36). An alternative to peptide stimulation is use of the anthrax toxin LFn, which has the ability to deliver full-length antigen into the cytosol for native processing via the major histocompatibility complex (MHC) pathways and the ability to elicit better T cell responses than peptides in some cases (37–42). Our adaptation of the LFn ELISPOT test allowed not only detection of human DENV and ZIKV infections but also characterization of the associated T cell responses to structural and nonstructural proteins. We demonstrated that individuals with pDENV and sDENV infections had similar IFN-γ and TNF-α T cell response patterns, with high cross-reactivity to ZIKV NS3 helicase but low cross-reactivity to the ZIKV structural proteins. A small number of individuals with sDENV infections had cross-reactive T cell responses to ZIKV structural proteins. Interestingly, however, individuals with pZIKV and ZIKVwpDENV infections had similar IFN-γ and TNF-α T cell response patterns, with strong responses to structural and nonstructural proteins. It is noteworthy that we observed comparably strong T cell responses to the structural proteins in pZIKV and ZIKVwpDENV cases, in contrast to Grifoni et al. (10), suggesting that the most recent infection in a given case may dictate the T cell response. Another possibility that cannot be excluded is represented by the differences in T cell stimulation strategies, which may be contributing to the observed differences. Additionally, due to the limited collection of blood samples from each patient, we were unable to distinguish CD4-specific responses from CD8-specific responses. Future characterization studies of CD4 and CD8 T cells by use of the LFn delivery system will be important.

Our study of ZIKV seroprevalence in West Africa demonstrated continued human transmission of the virus in HIV-infected and malaria-infected individuals (43). The coinfection of flaviviruses with HIV or malaria could potentially impact pathophysiological mechanisms, result in different clinical and laboratory findings, and interfere with treatment. Previous studies have shown suppression of HIV-1 replication during acute DENV infection (44, 45). In this study, we demonstrated that DENV-exposed individuals who were HIV infected had T cell responses whose levels were significantly lower than those shown by HIV-negative individuals except in individuals with pZIKV infections. We also observed that DENV-exposed HIV-infected individuals showed lower T cell responses to ZIKV proteins than DENV-exposed HIV-negative individuals. Of note, we did not observe significant differences in the sizes of IFN-γ and TNF-α spots in assays of individuals with multiple flaviviral infections or of those that were HIV infected versus those that were HIV negative. The issue of whether HIV infection in DENV-exposed individuals reduces the ability to induce cross-reactive T cell responses has important implications. More studies with larger sample sizes are needed to increase our limited understanding of the epidemiological and immunopathogenesis interactions of flavivirus exposure in individuals with HIV and other comorbidities.

In summary, despite high sequence homology between DENV and ZIKV, diagnostic assays based on antibodies to NS1 and T cell responses to NS3 protease are effective at distinguishing human infections by these viruses. The LFn ELISPOT test has enabled direct comparisons of results of T cell characterization in human infections by DENV and Asian and Africa ZIKV. As vaccines against DENV and ZIKV are currently being developed, the information generated from these characterization studies is of high relevance. The results of these characterization studies may contribute to the design and development of DENV and ZIKV vaccines and T cell-based diagnostics.

## MATERIALS AND METHODS

### Clinical samples and ethical statement.

Fifty late-convalescent-phase blood samples were obtained from patients at Edgard Santos University Hospital, Federal University of Bahia, Salvador, Brazil. These individuals were suspected to have been infected by ZIKV during the 2015–2016 ZIKV epidemic, and their acute-phase sera were screened for ZIKV and DENV antibodies. Late-convalescent-phase peripheral blood mononuclear cells (PBMCs) were separated from whole blood in EDTA tubes by Ficoll-Hypaque gradient density (Sigma-Aldrich, St. Louis, MO, USA) and cryopreserved in freezing media (10% dimethyl sulfoxide [DMSO], Sigma-Aldrich, St. Louis, MO, USA) at −80°C overnight prior to transfer to liquid nitrogen. Convalescent-phase serum was divided into aliquots and immediately transferred to −80°C.

The Federal University of Bahia Institutional Review Board (IRB), the Harvard T.H. Chan School of Public Health IRB, and the University of Hawaii IRB approved the primary studies under which the samples and data were collected. All patients provided informed consent for the collection of samples. Excess samples and corresponding data were banked, coded prior to analyses, and stored at the Federal University of Bahia.

### ELISAs.

For acute-phase sera, commercial ZIKV-NS1- and DENV-E-based IgG ELISAs (Euroimmun, Luebeck, Germany) were performed (27). For late-convalescent-phase sera, ZIKV- and DENV1-NS1 IgG ELISAs were performed as described previously (22). Briefly, purified NS1 proteins (16 ng per well) were coated onto 96-well plates overnight, followed by blocking and incubation with primary antibodies (serum at 1:400 dilution) and secondary antibodies (anti-human IgG conjugated with horseradish peroxidase [HRP]; Jackson) (22). The OD at 450 nm was read with a reference wavelength of 650 nm. Each ELISA plate included two positives (two confirmed-Zika and confirmed-dengue samples for ZIKV- and DENV-NS1 ELISAs, respectively), four negatives (4 flavivirus-naive serums), and tested samples (all in duplicates). The OD values were divided by the mean OD value of positive controls to calculate the rOD values. The cutoff was defined by the mean rOD value of negatives plus 12 standard deviations as described previously (22). For samples positive for both ZIKV- and DENV-NS1 ELISAs, the ratio of rOD (rOD ratio = rOD of ZIKV-NS1/rOD of DENV-NS1) was calculated; a rOD ratio value of <0.24 or ≥0.24 indicated sDENV or ZIKVwpDENV infection, respectively (22).

E protein-based IgG ELISAs using DENV1 virion or ZIKV (MR766 strain) virus-like particles (VLP) were also performed for late-convalescent-phase sera (46). Briefly, DENV1 virions or ZIKV VLP derived from ultracentrifugation of culture supernatants of virus-infected Vero cells or pENTR-ZIKV prME plasmid-transfected 293T cells, respectively, were UV inactivated (for virions) and coated on 96-well plates at 4°C overnight, followed by blocking and incubation with primary (serum at 1:800 dilution) and secondary antibodies as described above. The rOD and cutoff rOD values were similarly calculated. The difference in rOD of ZIKV and DENV E proteins (ΔrOD = rOD of ZIKV − rOD of DENV) was determined; ΔrOD values of greater than or equal to 0.17 or less than −0.17 were classified as representative of pZIKV or pDENV infection, respectively.

### Neutralization test.

PRNT was performed on acute-phase sera to detect neutralization antibody to ZIKV as reported previously (47). For late-convalescent-phase sera, a previously described microneutralization test was performed (48). Briefly, flat-bottom 96-well plates were seeded with Vero cells (3 × 10^4^ cells per well) 24 h prior to infection. Fourfold serial dilutions of serum (starting from 1:10) were mixed with 50 focus-forming units of DENV1 (Hawaii strain), DENV2 (NGC strain), DENV3 (CH53489), DENV4 (H241 strain), or ZIKV (PRVABC59 strain) at 37°C for 1 h. The mixtures were added to each well followed by incubation for 48 h (except 70 h for DENV1), removal of medium, and fixation as described previously (46). After adding a murine monoclonal antibody (MAb) 4G2 and secondary antibody mixture (IRDye 800CW-conjugated goat anti-mouse IgG at 1:10,000 and DRAQ5 fluorescent probe at 1:10,000), the signal (800 nm/700 nm fluorescence) was detected by the use of a Li-Cor Odyssey Classic imaging system (Li-Cor Biosciences) and analyzed using Image Studio software to determine percent neutralization at different concentrations and NT_90_ as described previously (46, 48).

### LFn fusion protein design.

Commercially synthesized gene fragments encoding the NS3 protease and helicase of DENV2 and the C, prM, and NS3 protease and helicase of Asian ZIKV were cloned into the LFn expression vector (pET15bLFn). The pET15bLFn vector contains a T7 promoter, a six-histidine tag (His_6_), and the terminal domain of the anthrax lethal factor (LFn; 255 amino acids). The pET15bLFn containing the coding sequences of the DENV and Asian ZIKV proteins were transformed into Escherichia coli BLR (DE3) (Millipore, Medford, MA). Selected clones were sequences used to verify the reading frame, and clones containing the correct sequence were used for protein expression.

The LFn-DENV and LFn-ZIKV fusion proteins and the LFn control were expressed upon induction of isopropyl-β-d-thiogalactopyranoside (IPTG; Sigma-Aldrich, St. Louis, MO, USA) in 5 liters of Luria broth containing carbenicillin and chloramphenicol for 2 to 4 h. Cells were pelleted by centrifugation and resuspended in imidazole (1 mM) binding buffer (Novagen, Madison, WI) in the presence of a protease inhibitor cocktail (Thermo Fisher Scientific, Rockford, IL). Cell pellets were sonicated and centrifuged at 4°C, and the supernatants were loaded in an equilibrated nickel-charged column for affinity purification. The bound proteins were eluted in 100 to 200 mM imidazole, desalted with a Sephadex G-25M column (Sigma-Aldrich, St. Louis, MO, USA), and eluted in phosphate-buffered saline (PBS) (Sigma-Aldrich, St. Louis, MO, USA). The PBS-eluted proteins were passed through Detoxi-Gel (Thermo Fisher Scientific, Rockford, IL). Protein concentrations were determined, and samples were stored at −80°C.

### ELISPOT test.

*Ex vivo* ELISPOT tests were performed as previously described. Briefly, 96-well polyvinylidene difluoride (PVDF)-backed MultiScreen_HTS_ (MSIP) microtiter plates (Millipore, Medford, MA) were treated with 100 µl of 90% ethanol for 30 s and washed 5 times with sterile PBS. Plates were coated with 100 µl of each capture antibody (Ab) mixed with PBS. Plates containing capture Abs were incubated overnight at 4°C. Plates were then blocked with 1% bovine serum albumin (BSA; Sigma-Aldrich, St. Louis, MO, USA)–PBS and washed 6 times with PBS. Cryopreserved PBMCs were thawed in R10 medium and incubated overnight at 37°C. PBMCs were washed 2 times with PBS and seeded at 2 × 10^5^ cells/well in a final volume of 100 µl/well. LFn-DENV and LFn-ZIKV proteins were added to each well. As a positive control, PBMCs were stimulated with phytohemagglutinin (PHA; Sigma-Aldrich, St. Louis, MO, USA). As a negative control, wells received LFn. After incubation for 24 to 28 h at 37°C in 5% CO_2_, the cells were discarded and plates were washed 3 times with PBS and 3 times with PBS–0.05% Tween 20 (PBST; Bio-Rad Technologies, Hercules, CA) to remove cells. The detection antibodies were added, and plates were incubated overnight at 4°C. Plates were then washed 6 times with PBST and then incubated for 2 h at room temperature with mixtures containing the enzymatic conjugates. To develop spots, plates were washed 4 times with PBST, 3 times with PBS, and 1 time with water. Vector Blue substrate solution (Vector Laboratories, Burlingame, CA) was added for 5 to 15 min before rinsing with water and air drying. Digitized images were analyzed for spots using a CTL immunosorbent spot reader (Cellular Technology Limited, Cleveland, OH, USA). DENV and ZIKV spots were calculated by subtracting the mean of the negative-control value from the mean value of the specific stimulation. Positive responses had to be greater than 4 times the mean background and 3 standard deviations above the background, with ≥55 spot-forming cells (SFC)/10^6^ PBMCs.

### ROC analysis.

The ELISPOT tests were validated using PBMCs from individuals who were confirmed by ELISA and/or neutralization tests to be DENV infected and/or ZIIV infected. Values of ratios of DENV and ZIKV NS3 protease to helicase were calculated, resulting in normalized test ratios (ZIKV NS3 protease divided by DENV NS3 protease) ranging from 0.15 to 2.95. On the basis of these data, we determined the optimal cutoffs between 0.15 and 2.95 by calculating the sensitivity (number of true positives divided by total confirmed positive values) and specificity (number of true negatives divided by the total confirmed negative values) at increments of 0.05 to the theoretical cutoff values. After calculation of the sensitivity and specificity values, the optimal cutoffs were defined as the highest sum of sensitivity and specificity such that the optimal cutoff values reflected the optimal sensitivity and specificity. The optimal cutoff values obtained for IFN-γ and TNF-α were 1.05 and 1.048, respectively (Prism 7; GraphPad Software, Inc., San Diego, CA).

### Multiple-sequence alignment and percent homology analysis.

Multiple-sequence alignment of DENV1 to DENV4 and ZIKV NS3 was performed using the Clustal Omega program (EMBL-EB, Cambridgeshire, United Kingdom). Averages for DENV and ZIKV NS3 protease and helicase proteins were calculated using the ExPASy Bioinformatics Resource Portal (Swiss Institute of Bioinformatics, Lausanne, Switzerland) on the basis of averages of the different homology values in the four DENV serotypes and ZIKV. Average conservation was determined on a per-residue basis for NS3 protease, helicase, and full-length protein.

### Statistical analysis.

Statistical analysis was performed using Prism 7 (GraphPad Software, Inc., San Diego, CA). Where appropriate, data were expressed as geometric positive means on box whisker and bar graphs ± standard deviation. Data comparisons were conducted using the Wilcoxon rank-sum test. A threshold *P* value of <0.05 was considered statistically significant.

### Data availability.

All relevant data have been included in the manuscript. We will provide any additional data upon request.
