# Erratum for Herrera et al., “T Cell Responses to Nonstructural Protein 3 Distinguish Infections by Dengue and Zika Viruses”

**DOI:** 10.1128/mBio.01995-18

**Published:** 2018-10-23

**Authors:** Bobby Brooke Herrera, Wen-Yang Tsai, Carlos Brites, Estela Luz, Celia Pedroso, Jan Felix Drexler, Wei-Kung Wang, Phyllis J. Kanki

**Affiliations:** a Department of Immunology and Infectious Diseases, Harvard T. H. Chan School of Public Health, Boston, Massachusetts, USA; b Department of Tropical Medicine, Medical Microbiology, and Pharmacology, John A. Burns School of Medicine, University of Hawaii at Manoa, Honolulu, Hawaii, USA; c Laboratory of Infection Research, School of Medicine, Federal University of Bahia, Salvador, Brazil; d Charité Universitatsmedizin Berlin, Freie Universität Berlin, Humboldt-Universität zu Berlin; e Berlin Institute of Health, Institute of Virology, Berlin, Germany; f German Centre for Infection Research, Bonn, Germany

## ERRATUM

Volume 9, issue 4, e00755-18, 2018, https://doi.org/10.1128/mBio.00755-18. We inadvertently referred to ZIKVwpDENV as ZIKV with primary DENV infection, instead of ZIKV with previous DENV infection, in the abstract and introduction (on PDF page 2, second paragraph). In addition, we had typos listing reference 22 rather than reference 12 in the text of Results (on PDF page 3, second paragraph) and Discussion (on PDF page 9, second paragraph). The affiliation lines should also have appeared as shown in this erratum. These changes do not affect the conclusions of the paper. We apologize for any confusion that might have arisen from our mistake.

