# Supplementary material for: T Cell Responses to Nonstructural Protein 3 Distinguish Infections by Dengue and Zika Viruses
Source: mBio. 2018 Aug 7;9(4):e00755-18. doi: 10.1128/mBio.00755-18 (PMC6083909; doi:10.1128/mBio.00755-18)
Supplement: TABLE S1 [file mbo004184017st1.docx]

**SUPPLEMENTARY TABLE**

**Supplementary Table 1** Cohort characteristics

|  | Characteristics | | |
| --- | --- | --- | --- |
| Patient ID | Sex | Age | HIV  status |
| ZK0978 | F | 49 | - |
| ZK0982 | F | 50 | - |
| ZK0987 | F | 33 | - |
| ZK0999 | F | 49 | + |
| ZK0979 | F | 28.9 | - |
| ZK0993 | M | 27 | + |
| ZK0998 | M | 55 | + |
| ZK1006 | F | 51 | + |
| ZK0996 | F | 23 | - |
| ZK0966 | M | 34 | + |
| ZK0980 | F | 33 | - |
| ZK0995 | M | 42 | + |
| ZK0997 | F | 37 | - |
| ZK0972 | F | 41 | + |
| ZK0975 | F | 67 | + |
| ZK0989 | M | 42 | + |
| ZK0991 | F | 24 | - |
| ZK1000 | M | 56 | + |
| ZK1009 | M | 52 | + |
| ZK1011 | M | 72 | + |
| ZK1012 | F | 40 | + |
| ZK1014 | F | 41 | - |
| ZK1015 | M | 54 | + |
| ZK0968 | M | 46 | + |
| ZK0984 | F | 58 | + |
| ZK0976 | F | 50 | + |
| ZK0986 | F | 53 | + |
| ZK0967 | F | 41 | + |
| ZK0969 | M | 32 | + |
| ZK0971 | F | 29 | + |
| ZK0977 | M | 52 | + |
| ZK0983 | F | 43 | + |
| ZL0985 | F | 33 | + |
| ZK0988 | M | 40 | + |
| ZK0992 | M | 53 | + |
| ZK0994 | F | 42 | + |
| ZK1001 | M | 40 | + |
| ZK1010 | M | 50 | + |
| ZK1013 | F | 31 | - |
| ZK0973 | M | 62 | + |
| ZK0974 | M | 39 | + |
| ZK1002 | M | 35 | + |
| ZK1003 | F | 57 | + |
| ZK1007 | F | 48 | + |
| ZK1016 | M | 46 | + |
| ZK0990 | F | 45 | - |
| ZK1004 | M | 28 | - |
| ZK1005 | F | 53 | + |
| ZK1008 | M | 62 | + |
| ZK0981 | F | 47 | - |
